# A novel mutation in CNNM4 is associated with a case of Jalili syndrome in Egypt

**DOI:** 10.1007/s10633-025-10018-1

**Published:** 2025-04-15

**Authors:** Caroline Atef Tawfik, Haneen Sabry Aly, Menna Kabeel, Iman Yousri, Sara Abdallah Mohamed

**Affiliations:** 1 Department of Ophthalmology, Ain Shams University, Cairo, Egypt; 2 Watany Eye Hospital, Cairo, Egypt; 3 Serenity Dental Clinic, Cairo, Egypt; 4 Department of Pediatrics, Cleopatra Hospitals Group, Cairo, Egypt

**Keywords:** *CNNM4*, Jalili syndrome, Syndromic cone-rod dystrophy, Novel mutation, Amelogenesis imperfecta

## Abstract

**Purpose:**

To report a novel homozygous mutation in *CNNM4* gene associated with Jalili syndrome (JS) which is a rare, recessively inherited oculo-dental syndrome which encompasses cone-rod dystrophy (CORD) and amelogenesis imperfecta (AI).

**Methods:**

A 4-year-old male patient of consanguineous Egyptian parents, who presented with progressive visual impairment and tooth decay underwent complete ophthalmological examination, dental, and systemic examination. Additionally, color fundus photography, fundus autofluorescence (FAF), spectral domain optical coherence tomography (SD-OCT) of the macula, full field electroretinogram (ffERG) were obtained. Orthopantomogram (OPG) were also obtained. NGS-based gene panel testing was done in a commercial laboratory from a peripheral blood sample.

**Results:**

Fundus examination demonstrated typical features of CORD in the form of loss of foveal reflexes with macular retinal pigment epithelial mottling and atrophy reminiscent of bull’s eye maculopathy. Dental assessment revealed evidence of AI. NGS-based gene panel identified a novel mutation in *CNMM4* gene c.1423 G>A consistent with a diagnosis JS, thereby confirming the rare diagnosis.

**Conclusion:**

To the best of our knowledge, this is the first report of Jalili syndrome in Egypt. We are reporting a novel mutation in *CNMM4* gene. We are also expanding the clinical spectrum of dental manifestation by reporting early eruption of the first permanent molars and suggesting that hyperopia could be a rather constant feature of JS. This case emphasizes the importance of comprehensive multidisciplinary assessment beyond visual complaints in IRD patients in order to reach an accurate diagnosis.

## Introduction

Jalili Syndrome (JS; MIM# 217080) is a rare, recessive disorder characterized by cone-rod dystrophy (CORD) and amelogenesis imperfecta (AI). First described in 1988 by Jalili & Smith who observed this new phenotype in 29 members of a consanguineous family from Gaza strip, where all affected members had photophobia, nystagmus, color vision defects along with abnormal tooth enamel [1]. With less than 150 cases reported up to date, this exceedingly rare syndrome has been reported mostly in the Middle East and North Africa [2, 3] with fewer reports worldwide [4]. Apart from the oculo-dental phenotype, there has been reports of other systemic findings including cognitive impairment [5], myopathy with muscular overgrowth [6], and keratoconus with situs inversus [7].

CORDs (MIM # 120970) are a group of progressive, clinically and genetically heterogenous inherited retinal diseases (IRDs) manifesting in childhood or early adulthood. Early in the course of the disease, they exhibit cone dysfunction presenting with central visual impairment, nystagmus, photophobia, color vision defects, and maculopathy. In advanced stages of the disease, patients often complain of night vision difficulties along with peripheral visual field defects denoting rod photoreceptor affection [8].

AI encompasses a group of genetic disorders affecting dental enamel composition and structure in both primary and permanent dentition. It is classified into two types namely: hypoplastic and hypomineralized AI. The former is essentially a quantitative enamel defect occurring in the secretory developmental stage, characterized by missing or thinned out areas of enamel with otherwise normal structure of teeth. The latter is a qualitative defect of enamel where it is of normal thickness with posteruptive breakdown. AI generally manifests as small, yellow to dark brown, pitted or grooved, and frangible teeth [9, 10].

*CNNM4* gene (MIM # 607805) formed of seven exons and located on chromosome 2q11.2 belongs to a family of highly conserved proteins. They were initially called ancient conserved domain proteins (ACDP). *CNNM4* has a structure comprised of four domains: extracellular domain, a transmembrane domain also known as domain of unknown function 21 (DUF21), cyclin and cystathionine ß-synthase (CBS) domain and a cyclic nucleotide-binding homology domain [4, 11, 12]. It encodes a transmembrane protein (CNNM4) expressed in retina and developing teeth. Its main role is in ion regulation mainly that of magnesium where it transports magnesium outside cells in exchange for extracellular sodium, hence playing a critical role in cellular functions related to metalloenzymes and transcription regulation [13–15].

Here we describe a 4-year-old male with JS caused by a novel homozygous mutation NM_020184.4: c.1423G>A, (p. Val475Met) in *CNNM4* gene and presenting with CORD and AI. To the best of our knowledge, this is the first report of JS in Egypt.

## Clinical report

A 4-year-old patient from a consanguineous Egyptian family presenting with visual impairment along with dental problems underwent ophthalmological, dental examination, and general examination. The study adhered to the tenets of the Declaration of Helsinki, where an informed, written consent was obtained from the guardians after explanation of the study and its purpose.

### Ophthalmic examination and investigations

The patient underwent ophthalmological examination including recording of ocular and family histories, refraction, as well as slit lamp biomicroscopy and a dilated fundus examination.

Ultra-wide field fundus photography and fundus autofluorescence (FAF) using Optos, California (Optos, Malborough, MA, USA), spectral domain optical coherence tomography (SD-OCT) using Heidelberg Spectralis OCT (Heidelberg Engineering GmbH; Dossenheim, Germany) of the macula were obtained. Additionally, full field electroretinogram (ffERG) was recorded via HK-loops using the Sunburst Ganzfeld stimulator (UTAS Sunburst; LKC Technologies, Gaithersburg, MD, USA) according to the International Society for Clinical Electrophysiology (ISCEV) standards.

### Dental examination and investigations

The patient underwent a detailed dental assessment including a thorough extraoral and intraoral examination.

Frontal, upper and lower occlusal intraoral photographs were taken and orthopantomograms (OPG) were obtained using PaX-i Insight device (Vatech Co., Hwaseung Si, Korea).

### Systemic/neurological examination

A head-to-toe general examination with assessment of overall general health and development was done with emphasis on neurological examination. Laboratory investigations in the form of complete blood count (CBC), electrolyte assessment (calcium and magnesium) and vitamin D assay were ordered. An echocardiography was also performed to assess the rare association of situs inversus.

### Molecular genetic analysis

We performed genetic testing via an extensive, NGS-based gene panel on an Illumina platform in a commercial laboratory by using genomic DNA extracted from a peripheral blood sample.

## Results

### Ophthalmological findings

Visual acuity couldn’t be assessed properly due to the patient’s young age and lack of cooperation. His cycloplegic refraction was hyperopic with + 7.00 D sphere in both eyes.

**Fig. 1:**
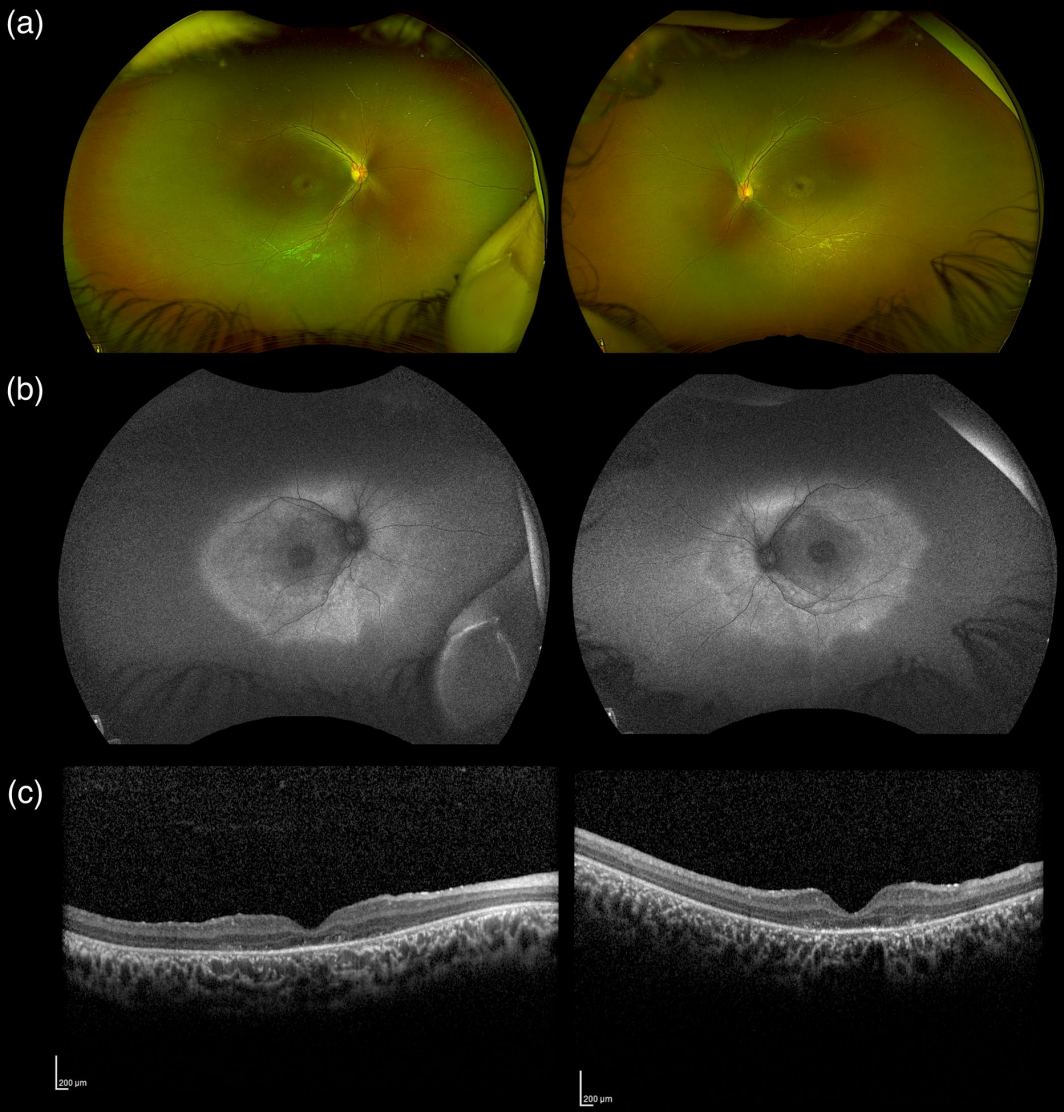
Multimodal imaging of our patient**. a** Ultra-wide field pseudocolor fundus image of the right and left eye respectively demonstrating macular atrophy with a bull’s eye maculopathy with mildly attenuated blood vessels. **b** Ultra-wide field green light short-wavelength fundus autofluorescence image of the right and left eye respectively demonstrating markedly decreased macular autofluorescence with a surrounding ring of hyperautofluorescence just outside the arcades. (c) OCT of the macula showing foveal outer retinal atrophy with diffuse thinning

Fundus examination and UWF imaging demonstrated typical features of CORD in the form of loss of foveal reflexes with macular retinal pigment epithelial mottling and atrophy reminiscent of bull’s eye maculopathy. UWF-FAF showed markedly decreased macular autofluorescence with a surrounding ring of hyperautofluorescence outside the arcades. OCT showed decreased foveal thickness, ellipsoid zone disruption with outer retinal layers atrophy mainly in the subfoveal area (Fig. 1).

ffERG showed non-recordable cone responses with 65–75% reduction in rod responses thereby confirming the diagnosis of CORD.

### Dental findings

**Fig. 2:**
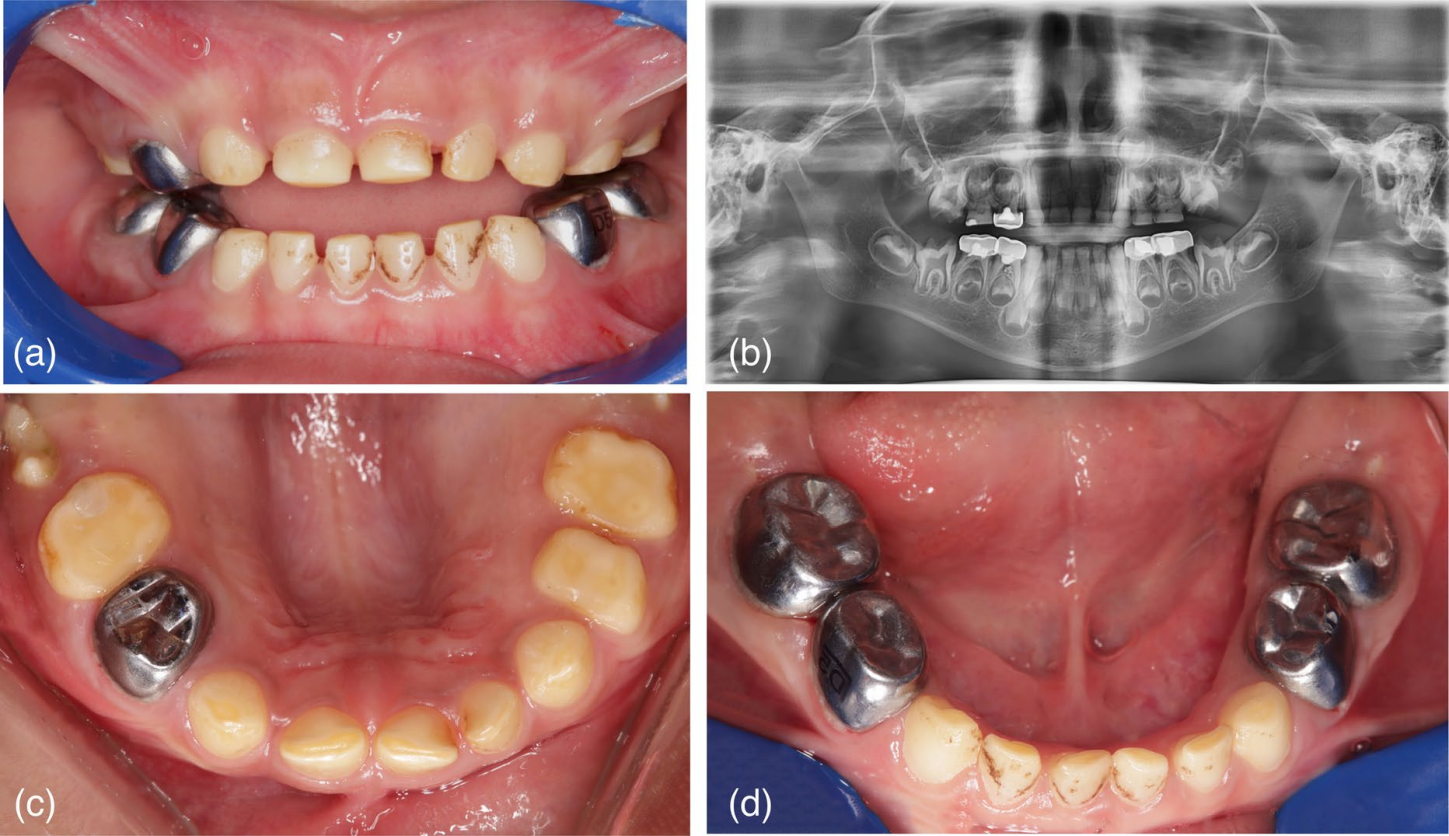
Dental findings of our patient. **a** Intraoral frontal photograph showing upper and lower arches in primary dentition. Generalized gingivitis and plaque accumulation are evident. Anterior primary teeth are hypoplastic, discolored showing pitting and chromogenic stains. Increased vertical occlusal height is evident with ill-fitting stainless-steel crowns. **b** Extraoral orthopantomogram showing previously treated deciduous molars, hypoplastic erupting first permanent molars with enlarged pulp cavities and obliterated pulp canals of anterior deciduous teeth. Generalized reduction in enamel thickness involving both primary and unerupted permanent dentition. **c** Intraoral occlusal photograph of upper arch showing primary dentition. Gingiva exhibits signs of mild inflammation and plaque accumulation. Erupting first permanent molars showing hypoplasia and discoloration that also involves all deciduous teeth. Carious lesions evident in upper right central and lateral incisors and primary first molars. **d** Intraoral occlusal photograph of lower arch showing primary dentition. Gingiva exhibits signs of mild inflammation and plaque accumulation. Multiple stainless-steel crowns covering deciduous molars. Anterior teeth showing discoloration, hypoplasia, pitting and mild attrition

Dental examination revealed visibly discolored, erupting buccal cusps of the upper first permanent molars with an early eruption pattern. The primary teeth exhibited reduced size, staining, and loss of contact, with yellow discoloration, enamel pitting and reduced enamel quantity, indicative of AI. Additionally, occlusal interference from improperly positioned crowns has resulted in an open bite with generalized anterior spacing. **(**Fig. 2a, c and d).

OPG findings demonstrated severe reduction in enamel thickness in the erupting first permanent molars and the lower unerupted premolars indicative of hypoplastic AI. The distinction between enamel and dentin was not discernible on the panoramic radiograph. Enamel loss was seen in all primary teeth accompanied with pulpal canal obliterations. Enlarged pulp cavities was evident in the erupting permanent first molars and no evidence of congenital tooth agenesis was noted (Fig. 2b).

### Systemic/neurological findings

**Fig. 3:**
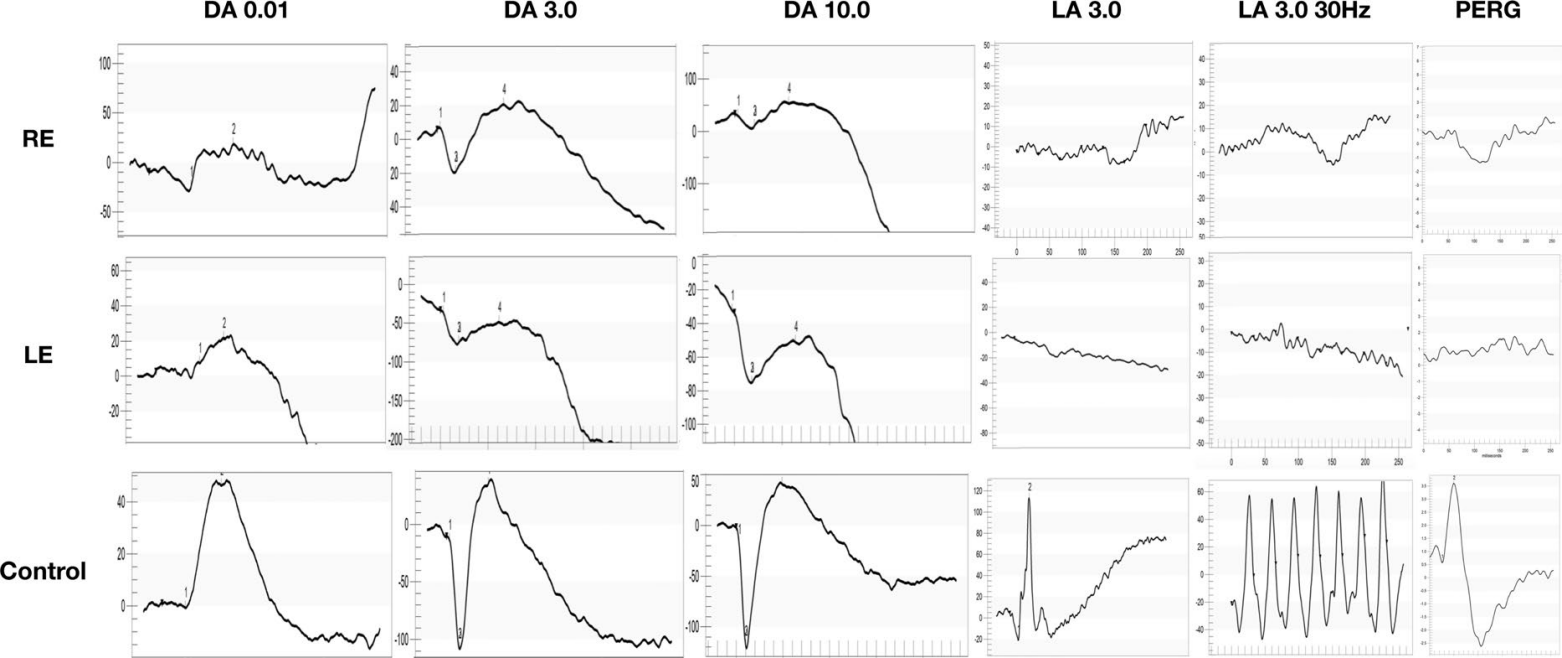
Electroretinogram of the patient. First three columns present dark-adapted full-field ERG (DA 0.01, 3.0, and 10.0), columns 4–5 present light-adapted (LA 3.0 and 3.0 30 Hz), and last column shows pattern ERG traces. Upper panel: right eye, middle panel: left eye, and lower panel: control

Patient’s weight and height were within normal along with normal neurological development. Review of other systems was unremarkable. His laboratory workup was within normal save for iron deficiency anemia and vitamin D deficiency, for which he received treatment. His echocardiography revealed no abnormalities (Fig. 3).

### Genetic findings

We identified a novel homozygous variant NM_020184.4: c.1423G>A, (p.Val475Met) in *CNNM4* gene. No other genes with potentially pathogenic variants were identified.

The canonical missense variant located in CBS domain was reported to be pathogenic with putative loss-of-function of the CNNM4 protein. This variant has very low frequency (< 0.01%) in control databases and was never reported in homozygous form. It was predicted to be deleterious (PP3) by all in silico prediction tools with a Revel score of 0.95. The variant allele identified has been deposited on ClinVar, with the following accession number: SCV005326512.

## Discussion

JS is a rare recessive oculo-dental syndrome that presents with both CORD characterized by childhood-onset poor vision and photophobia, and AI characterized by discolored, pitted, frangible teeth with caries. It is caused by bi-allelic mutations in *CNNM4* gene [2].

In the retina, CNNM4 function has been previously studied. It has been shown to interact with IQCB1 in the mouse photoreceptors which is essential for outer segment formation [16, 17]. In turn, IQCB1 interacts with CEP290 [18], calmodulin and forms a complex with RPGR [19]. Mutations in *CNNM4* gene may result in CORD and AI secondary to nonsense mediated mRNA decay, apoptosis and/or affection of IQCB1 function [16].

Regarding the ocular phenotype, the patient has presented with visual impairment and photophobia typical of cone system dysfunction which was confirmed by ERG. His refraction was highly hyperopic which is the most prevalent refractive error reported with JS [1, 6, 15, 20–26] with few exceptions [27, 28] which suggests that this could be a possible expansion of clinical spectrum of JS.

Regarding the dental phenotype, it was consistent with hypoplastic AI where he had discolored, pitted teeth with generalized reduction in enamel thickness. We are also reporting premature eruption of first permanent molars which has only been reported once before in the literature, suggestive of a novel feature expanding its clinical spectrum of AI [29]

Our variant c.1423G>A has not been reported previously as pathogenic, but it was predicted to be deleterious by in silico prediction tools. There have been no reports of patients presenting with both CORD and AI due to variants in other genes. Hence, the detailed clinical report presenting with typical phenotype supports further the pathogenicity of this novel variant.

We emphasize the role of deep phenotyping and proper assessment of systemic symptoms beyond visual complaints in IRD patients. We cannot stress enough on the importance of genetic testing in unraveling syndromic diseases specifically when systemic symptoms are not very obvious. Hence, a comprehensive approach highlights the need for multidisciplinary management of IRD and enables proper genetic counseling.

In conclusion, we expand the number of cases of JS as well as the genetic landscape of mutations associated with JS. We also suggest hyperopia and premature eruption of permanent dentition as expansion of the clinical spectrum. Future research directed towards understanding more about *CNNM4* gene function may lead to better correlation between genotype and phenotype.

## Data Availability

The authors confirm that the data supporting the findings of this study are available within the article [and/or] its supplementary materials.

## Declarations

### Statement on the welfare of animals

This study did not involve the use of animals. No animal subjects were included in the case reports, and no procedures were performed on animals. As such, ethical approval for animal research was not required.
